# Anthropogenic carbon pathways towards the North Atlantic interior revealed by Argo-O_2_, neural networks and back-calculations

**DOI:** 10.1038/s41467-024-46074-5

**Published:** 2024-02-22

**Authors:** Rémy Asselot, Lidia I. Carracedo, Virginie Thierry, Herlé Mercier, Raphaël Bajon, Fiz F. Pérez

**Affiliations:** 1 University of Brest, Ifremer, CNRS, IRD, Laboratory of Spatial and Physical Oceanography (LOPS), 29280 Plouzané, France; 2 Institute of Marine Investigations (IIM, CSIC), 6 Eduardo Cabello Street, 36208 Vigo, Spain

**Keywords:** Physical oceanography, Carbon cycle, Physical oceanography

## Abstract

The subpolar North Atlantic (SPNA) is a region of high anthropogenic CO_2_ (C_ant_) storage per unit area. Although the average C_ant_ distribution is well documented in this region, the C_ant_ pathways towards the ocean interior remain largely unresolved. We used observations from three Argo-O_2_ floats spanning 2013-2018 within the SPNA, combined with existing neural networks and back-calculations, to determine the C_ant_ evolution along the float pathways from a quasi-lagrangian perspective. Our results show that C_ant_ follows a stepwise deepening along its way through the SPNA. The upper subtropical waters have a stratified C_ant_ distribution that homogenizes within the winter mixed layer by Subpolar Mode Water formation in the Iceland Basin. In the Irminger and Labrador Basins, the high-C_ant_ footprint (> 55 μmol kg^−1^) is mixed down to 1400 and 1800 dbar, respectively, by deep winter convection. As a result, the maximum C_ant_ concentration is diluted (<45 μmol kg^−1^). Our study highlights the role of water mass transformation as a first-order mechanism for C_ant_ penetration into the ocean. It also demonstrates the potential of Argo-O_2_ observations, combined with existing methods, to obtain reliable C_ant_ estimates, opening ways to study the oceanic C_ant_ content at high spatio-temporal resolution.

## Abstract (web summary)

Large emissions of anthropogenic carbon dioxide have been partly absorbed by the oceans. Here, the authors use Argo-O2 floats combined with existing methods to study the distribution of this anthropogenic CO2 in the North Atlantic Ocean.

## Introduction

Since the beginning of the industrial revolution, human activities such as fossil fuel burning and changes in land-use have led to the emission of large amounts of carbon dioxide (CO_2_) into the atmosphere. This excess of carbon, referred to as anthropogenic carbon (C_ant_), represented an addition of 9.5 ± 0.5 GtC yr^−1^ to the atmosphere between 2011 and 2020^1^. The ocean has absorbed 25 ± 2% of C_ant_ emissions (170 ± 35 GtC out of 660 ± 65 GtC of total C_ant_ emissions) since the beginning of the industrial era^1^. The ocean acts, therefore, as a net C_ant_ sink and a moderator of climate change. This net C_ant_ uptake occurs via air-sea exchange, driven by air–sea CO_2_ disequilibria resulting from the difference between the steady increase in atmospheric *p*CO_2_ over the years since the pre-industrial era and oceanic *p*CO_2_^2^. The latter is primarily regulated by seasonal variations of temperature in the subtropics. In contrast, at higher latitudes, its oscillation is typically dominated by biological processes^3^ affecting dissolved inorganic carbon (DIC) and total alkalinity (A_T_). A low DIC/A_T_ ratio will lead to a large CO_2_ uptake capability by the ocean in response to an increase in atmospheric *p*CO_2_^4^. As a result of the accumulation of C_ant_, the Revelle factor, measuring the buffer capacity of the ocean, decreases^5^. Ultimately, changes in oceanic circulation also have the capacity to modify the saturation of C_ant_ at the ocean surface, enhancing or dampening the ocean carbon uptake capability^6^.

**Fig. 1:**
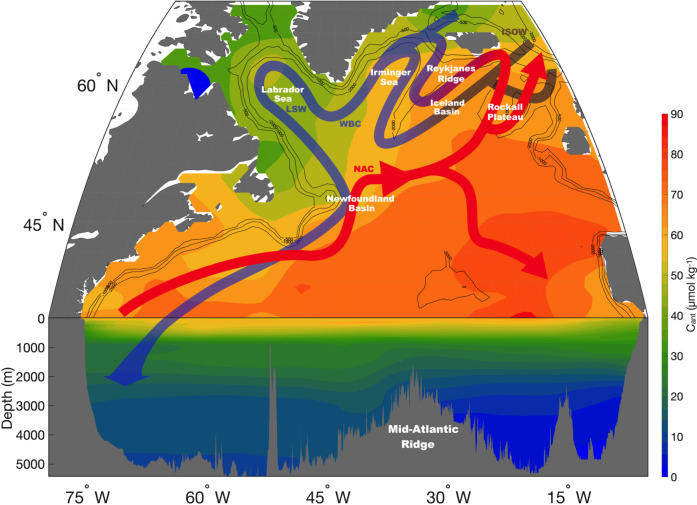
Main circulation patterns and C_ant_ distribution (μmol kg^−1^) for the year 2015 in the subpolar North Atlantic gyre. Red arrows represent the upper limb of the Atlantic Meridional Oceanic Circulation (AMOC) while blue arrows indicate the lower limb of the AMOC. The purple arrow represents ocean circulation between the upper and lower limb of the AMOC. The black contours represent the isobath −500 m, −1000 m, and −2000 m. The section on this figure depicts the whole water column, while our study focuses on the top 2000 m of the water column only. NAC North Atlantic Current, WBC Western Boundary Current, ISOW Iceland-Scotland Overflow Water, LSW Labrador Sea Water. C_ant_ concentrations come from the GLODAPv2 dataset^56^ and were normalized to the year 2015 using the exponential equation of Carter et al.^25^.

The global C_ant_ distribution in the ocean is not homogeneous, neither vertically nor horizontally^7^. As expected from direct air-sea exchange, C_ant_ concentration is maximum at the surface and decreases with depth. Of the global ocean, the North Atlantic Ocean (Fig. 1) is the region with the largest C_ant_ inventory per surface area^8,9^, storing up to 23–38% of the total oceanic C_ant_^2,10^. Within this region, C_ant_ uptake mostly occurs in the subtropics^6,7^ due to the low Revelle factor of subtropical surface waters^2^. These subtropical C_ant_-loaded waters are then transported northward toward subpolar latitudes^6,11^ by the upper branch of the Atlantic Meridional Overturning Circulation (AMOC), where additional C_ant_ uptake takes place^12^. The subpolar North Atlantic (SPNA) gyre has been identified as one of the most important regions for the injection of C_ant_ towards the deep ocean^7^. This deep C_ant_ penetration is mainly due to the formation of Labrador Sea Water (LSW) through deep winter convection in the Labrador and Irminger Seas^7,13–16^. Considering the role of the ocean in moderating the ongoing climate change through its capacity to uptake and store C_ant_, any change in its uptake and storage rates might have drastic consequences on the climate system. Changes in the C_ant_ storage rates in the North Atlantic Ocean are fundamentally related to the increased atmospheric *p*CO_2_, the changes in the AMOC strength, the changes in the North Atlantic Oscillation (NAO) index, and deep water formation^6,16,17^. The current C_ant_ increasing trends and related C_ant_ storage rates might thus be modified in the future due to the increasing atmospheric C_ant_, and the projected weakening of both AMOC^18^ and deep-water formation^19,20^.

To date, C_ant_ estimates are mainly based on methods that rely on scarce but valuable ship-based measurements of carbonate system parameters (carbon-based methods^2,6,21–23^) or transient tracers such as CFCs (transient tracer-based methods^9,16^). However, studying the spatio-temporal evolution of oceanic C_ant_ storage and understanding the processes involved is a crucial challenge that requires a more detailed view of the upper and deep C_ant_ distribution and of its main pathways into the ocean interior. Additionally, the distribution of C_ant_ on timescales shorter than GO-SHIP cruises is necessary to understand the effect of CO_2_ emissions reduction and carbon removal strategies on the ocean. Considering the unrivaled spatio-temporal sampling provided by the Argo-O_2_ network^24^, the purpose of this study is to demonstrate that Argo-O_2_ data combined with existing neural networks (i.e., ESPER_NN^25^, CANYON-B and its routine CONTENT^26^) and a back-calculation method (φC_T_^O^ method^17,27^) can be used to obtain reliable C_ant_ estimate at the finest spatio-temporal scale to date. As a case study, we selected three Argo-O_2_ floats to describe C_ant_ deepening as it enters and propagates along the SPNA region. We highlight the long journey of C_ant_ from the surface towards the interior of the SPNA gyre. Our results open up perspectives to investigate C_ant_ storage changes and transport at seasonal to decadal timescales with the growing global and seasonally unbiased Argo-O_2_ data network compared to ship-based measurements.

## Results

### Distribution of C_ant_ associated with the North Atlantic Current

**Fig. 2:**
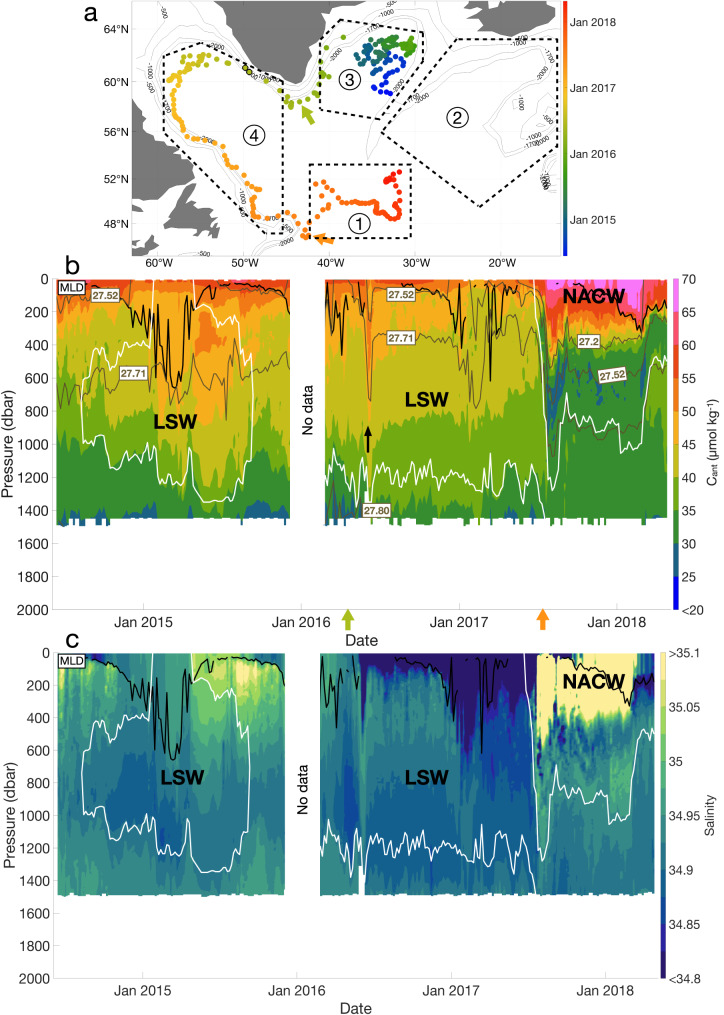
Characteristics of the Argo float 5904988 in the North Atlantic (47-65°N; 15-65°W). **a** Trajectory of the float with the four main regions defined. The color coding represents the date, with blue being the beginning of the trajectory and red the end. The green arrow indicates the moment the float passes Cape Farewell, and the orange arrow indicates when the float enters Region 1. The points with black outlines correspond to the Argo profiles identified as belonging to a passing eddy (see main text). **b** Section of estimated anthropogenic carbon (μmol kg^−1^) along the float pathway. The black line represents the mixed layer depth (MLD). The white lines represent the limits of the Labrador Sea Water (LSW), defined by O_2_ ≥ 290 μmol kg^−1^ in the Labrador and Irminger Seas and by *S* < 34.94 outside these two basins. The brown lines represent the isopycnals (kg m^−3^). The black arrow indicates when the float is located within an eddy. LSW Labrador Sea Water, NACW North Atlantic Central Water. **c** Salinity section along the float pathway.

Four main regions with the different vertical distributions of C_ant_ were identified along the floats’ pathways: region 1, located East of the Grand Banks of Newfoundland, and regions 2, 3, and 4, comprising the Iceland, Irminger, and Labrador basins, respectively (Fig. 2a). Region 1 is located inside a dynamic transition zone separating the subpolar and subtropical domains of the North Atlantic Ocean^28^. This transition zone is typically characterized by a sharp thermohaline and density front, which, in this case (Fig. 2c), indicates that float 5904988 entered the North Atlantic Current (NAC)^29^. The thermohaline front is also accompanied by a sharp C_ant_ and oxygen gradient (Fig. 2b and Supplementary Fig. 5). In the first 400–600 dbar of the water column, region 1 is characterized by saline, warm, and poorly oxygenated North Atlantic Central Water (NACW) of subtropical origin. At this location, the NACW comprises the northern extension of the upper and thermocline subtropical waters, defined as water masses located between the surface and *σ*_θ_ = 27.0 kg m^−3^ ^12^. These waters are typically characterized by a low Revelle factor^5^, which favors atmospheric C_ant_ uptake along their journey from the subtropics. As a consequence, the NACW presents the highest C_ant_ values (maximum C_ant_ > 65.0 μmol kg^−1^) of all water masses identified along the pathway of float 5904988 (Fig. 2a). The values of C_ant,def_ close to zero (Supplementary Fig. 3) indicate that the NACW is saturated in C_ant_ due to the air-sea C_ant_ uptake occurring during the journey of the water masses that form the NACW. Furthermore, just eastward of the outcrop of the isopycnal 27.2 kg m^−3^, the high C_ant_ concentrations (C_ant_ > 55.0 μmol kg^−1^) reach the base of the winter mixed layer depth (MLD) at ~400 dbar (Fig. 2b). In this region, the high C_ant_ signal is vertically transferred as the MLD deepens, highlighting the vertical C_ant_ homogenization due to mixing during winter convection. Below the mixed layer, between August 2017 and March 2018, C_ant_ concentration in region 1 decreased sharply to a relative minimum of 26.2 ± 6.6 μmol kg^−1^ near 800 dbar (Fig. 2b). These concentrations increase again up to 36.1 ± 7.5 μmol kg^−1^ at 1050 dbar, associated with the Labrador Sea Water (LSW). As inferred by the progression of the float, the C_ant_-loaded NACW is transported northwards by the NAC (Fig. 1), toward the Iceland Basin (region 2), following the general circulation pattern of the SPNA gyre^30^.

### Distribution of C_ant_ in the Iceland Basin and over the Reykjanes Ridge

**Fig. 3:**
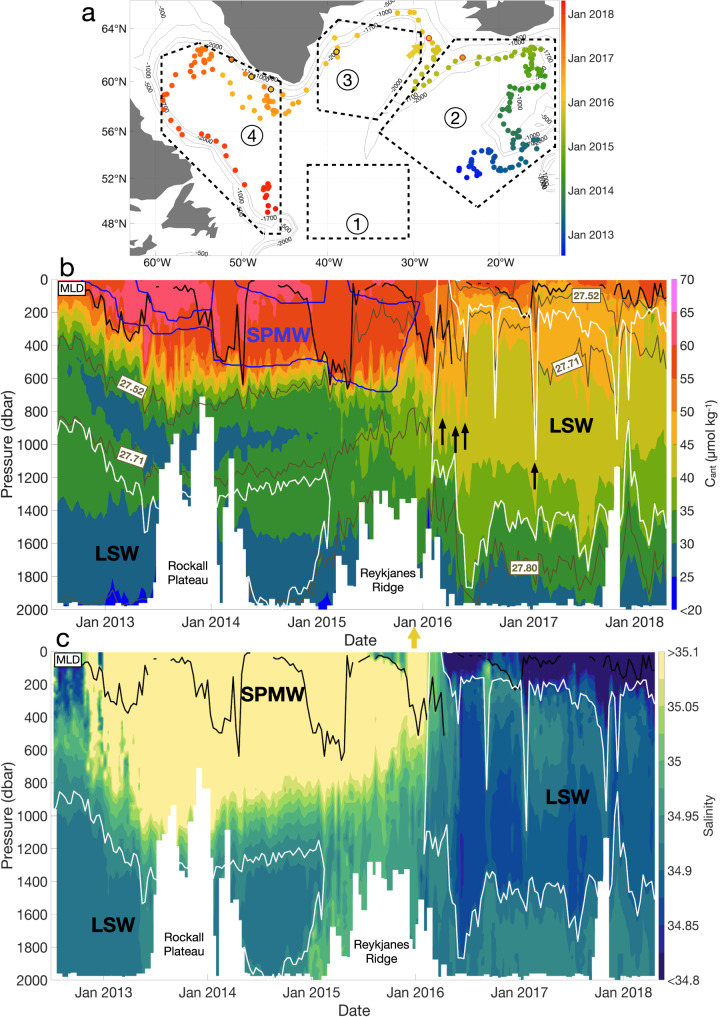
Characteristics of the Argo float 6901023 in the North Atlantic (47–65°N; 15–65°W). **a** Trajectory of the float with the four main regions. The color coding represents the date, with blue being the beginning of the trajectory and red being the end. The yellow arrow indicates when the float has passed the Reykjanes Ridge. The points with black outlines correspond to the profiles pointed out by the black arrows on the C_ant_ section. The points with red outlines represent the limits of the Reykjanes Ridge, defined by a depth shallower than 1700 m. **b** Section of C_ant_ (μmol kg^−1^) along the float pathway. The black line represents the MLD. The white lines represent the limits of the Labrador Sea Water (LSW), defined by O_2_ ≥ 290 μmol kg^−1^ in the Labrador and Irminger Seas and S < 34.94 outside these two basins. The blue lines represent the limits of the Subpolar Mode Water (SPMW) defined by a potential vorticity lower than 6 × 10^−11^ m^−1^ s^−1^. The brown lines represent the isopycnals (kg m^−3^). Black arrows indicate when the float is located in an eddy. **c** Salinity section along the float pathway.

**Fig. 4:**
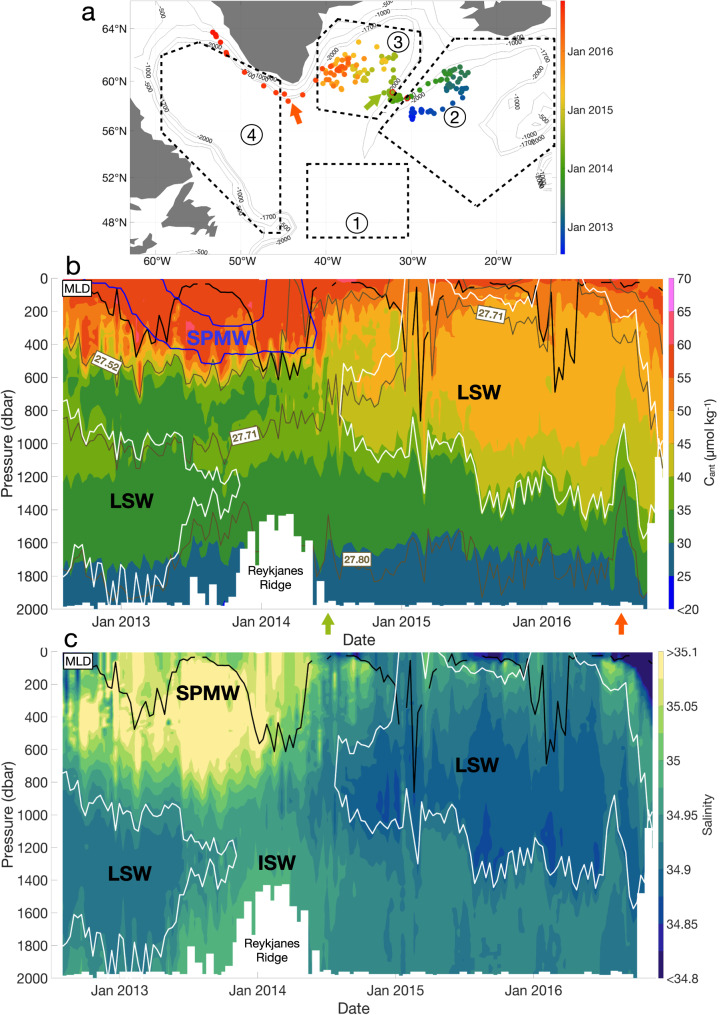
Characteristics of the Argo float 6901026 in the North Atlantic (47–65°N; 15–65°W). **a** Trajectory of the float with the four main regions. The color coding represents the date, with blue being the beginning of the trajectory and red being the end. The green arrow indicates when the float has passed the Reykjanes Ridge, while the red arrow shows when the float passes Cape Farewell. The points with red outlines represent the limits of the Reykjanes Ridge, defined by a depth shallower than 1700 m. **b** Section of C_ant_ (μmol kg^−1^) along the float pathway. The black line represents the MLD. The white lines represent the limits of the Labrador Sea Water (LSW), defined by O_2_ ≥ 290 μmol kg^−1^ in the Labrador and Irminger Seas and *S* < 34.94 outside these two basins. The blue lines represent the limits of the Subpolar Mode Water (SPMW) defined by a potential vorticity lower than 6 × 10^−11^ m^−1^ s^−1^. The brown lines represent the isopycnals (kg m^−3^). **c** Salinity section along the float pathway.

**Fig. 5:**
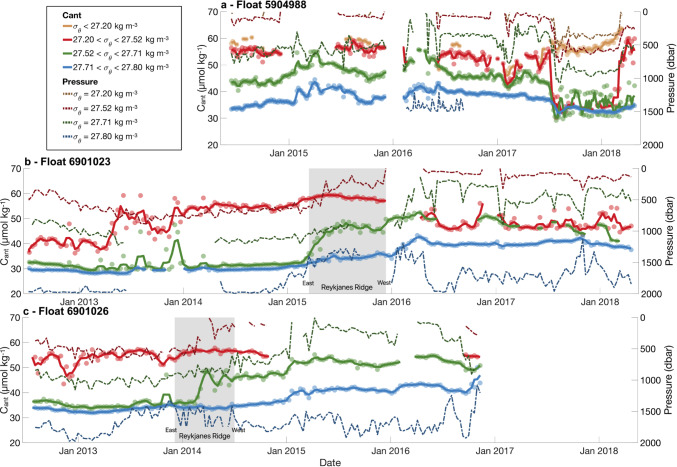
Averaged C_ant_ concentrations (μmol kg^−1^) over density layers. **a** For float 5904988. **b** For float 6901023. **c** For float 6901026. Scatter points indicate raw C_ant_ data, while the solid lines represent the monthly moving mean of C_ant_ concentrations. Dashed lines represent the pressure levels of the isopycnals. The gray rectangle illustrates the time period when the floats are above the Reykjanes Ridge.

Region 2 encompasses the Iceland Basin, including the eastern flank of the Reykjanes Ridge, and is characterized by the presence of Subpolar Mode Water (SPMW) in the first 600 dbar of the water column. In region 2, the SPMW contains the largest C_ant_ values over the water column, whose average is 57.2 ± 5.8 μmol kg^−1^. The SPMW is formed during winter convection^31,32^ by intense air–sea buoyancy loss, leading to the densification of the C_ant_-loaded NACW on its way toward subpolar latitudes^33^. This process is well illustrated by the good agreement between the winter MLD and the depth of the SPMW base (Figs. 3b and 4b). In the Iceland Basin, the depth of the isoC_ant_ 50 μmol kg^−1^, which mostly follows the winter MLD, reaches up to 600 dbar (Figs. 3b and 4b), that is, 200 dbar deeper than in region 1. Below the SPMW, C_ant_ concentration decreases rapidly, highlighting trapping of C_ant_ within the SPMW and revealing that the formation of this water mass is a precursor in the deepening of C_ant_ in the subpolar gyre. To further assess this deepening, Fig. 5 illustrates the mean C_ant_ concentration along four density ranges describing the main water masses found in the four regions^12,34^. In the second-density layer (27.2 < *σ*_θ_ < 27.52 kg m^−3^) of region 2, the averaged C_ant_ concentration along the float 6901023 pathway increases from 40.1 ± 6.2 μmol kg^−1^ in the south-easternmost part of the region to 55.5 ± 6.5 μmol kg^−1^ on the east side of the Reykjanes Ridge (Fig. 4b). This east-west C_ant_ enrichment is likely due to the trajectory of the float, going from an area containing a thin SPMW layer, to an area with a thick SPMW layer (Fig. 3b). In this region, C_ant,def_ fluctuates between −20 μmol kg^−1^ and 0 μmol kg^−1^ (Supplementary Fig. 3), suggesting that air–sea C_ant_ enrichment takes place, explaining part of the east–west C_ant_ enrichment in the Iceland basin. In the western part of Region 2, the Reykjanes Ridge entails a natural topographic barrier separating the Iceland and Irminger basins, two basins with different C_ant_ distribution and water masses. The isoC_ant_ 35 μmol kg^−1^, which fluctuated between 600 and 800 dbar east of the Reykjanes Ridge, deepens until ~1200 dbar on the western side of the ridge (Figs. 3b and 4b). The second density layer of region 2 (27.2 < *σ*_θ_ < 27.52 kg m^−3^) disappears west of the ridge during the winter months (Fig. 5, Supplementary Figs. 6c and 7c) due to outcropping and subsequent surface water densification in the vicinity of the ridge^12^. As a consequence, the third oceanic layer (27.52 < *σ*_θ_ < 27.71 kg m^−3^) shallows on the western side of the Reykjanes Ridge, illustrated by the shallowing of the isopycnal 27.71 kg m^−3^ from ~1000 dbar east of the ridge to less than 500 dbar west of the ridge (Fig. 5b, c). Along with this densification of the surface water, C_ant_ increases by 34% and 31% in the third layer for float 6901023 and float 6901026, respectively, marking a rapid transfer of C_ant_ into the third oceanic layer. However, if it cannot be excluded that the C_ant_ increase is only due to vertical entrainment, this increase could partly be due to lateral entrainment of water masses from another origin. These results highlight the role of the Reykjanes Ridge, separating the C_ant_ distribution and dynamics between region 2 and region 3^34^. In the fourth oceanic layer (27.71 < *σ*_θ_ < 27.80 kg m^−3^), C_ant_ concentrations are the lowest and slightly increase along the float trajectories (Fig. 5b, c). This increase stays within the averaged uncertainty range (±5.9 μmol kg^−1^) and is therefore not significant.

### Deep transport of C_ant_ in the Irminger and Labrador basins

The Irminger Sea (region 3) is characterized by high concentrations of O_2_ (Supplementary Figs. 5b, 6b, and 7b), delimiting the core of the LSW^34^. These large O_2_ values are indicative of the intense air-sea exchange that characterizes this region^13,35,36^. In contrast to regions 1 and 2, here, the vertical C_ant_ distribution has been subject to a large water-column homogenization. This leads to a weaker C_ant_ gradient, with a gradual decrease of mean C_ant_ concentrations from 52.0 ± 5.8 μmol kg^−1^ at the surface to 41.2 ± 7.9 μmol kg^−1^ at the lower bound of the LSW (~1400 dbar) (Figs. 2b and 4b). The high C_ant_ values are constrained within the LSW, meaning that the formation of this water mass traps and transports C_ant_ toward the deeper ocean. Yet, the maximum C_ant_ concentrations are still found at the surface, with values reaching 52.0 ± 5.8 μmol kg^−1^ (Figs. 2b and 4b). As suggested by the negative C_ant,def_ in the surface waters of the Irminger Sea (Supplementary Fig. 3b and 3c), these high surface concentrations are likely due to the intense air–sea CO_2_ exchange, following the same behavior of O_2_ exchanges^35^. Following the Western Boundary Current (WBC) (Fig. 1), C_ant_ is transported out of the Irminger Sea. In the WBC, we observe the occurrence of occasional high-C_ant_ pulses throughout the water column (black arrows on Figs. 2b and 3b) that we relate to anticyclonic mesoscale eddies (Supplementary Fig. 8). These anticyclonic features lead to a punctual downward isopycnal displacement of surface waters containing high C_ant_ concentration, explaining the C_ant_ pulses identified in our sections. Such eddies could carry C_ant_ away from their formation regions and when they collapse, they might contribute to the isopycnal mixing of this tracer. Once in the Labrador Sea, float 6901023 illustrates how the isoC_ant_ 35 μmol kg^−1^ reaches a maximum pressure level of 1800 dbar in the inner part of the basin (Fig. 3b). That is, implying a ~ 200 dbar deepening of this isoC_ant_ compared to what is observed in the Irminger Sea. As in the Irminger Sea, in the Labrador Sea, the high C_ant_ concentrations are also contained within the LSW, confirming that the formation of this water mass is a first-order deep-trapping mechanism for C_ant_.

## Discussion

This study describes the C_ant_ evolution along its pathways from the upper to the intermediate layers (up to 2000 dbar) of the SPNA gyre. It is based on a combination of existing neural networks and the back-calculation φC_T_^O^ method, using Argo-O_2_ observations as input data. Our results are based on three floats following the main branches of the subpolar gyre circulation. From this quasi-lagrangian perspective, we identified four main C_ant_ deepening stages and related them to their corresponding physical mechanisms. At the southernmost boundary of our study region, in the subtropical/subpolar transition zone near Flemish Cap, C_ant_ is mainly trapped within the NACW layer (*σ*_θ_ < 27.2 kg m^−3^). The NACW has the largest mean C_ant_ concentration (62.8 ± 5.9 μmol kg^−1^) of all water masses intercepted by the studied floats. At this location, winter mixing favors vertical C_ant_ homogenization, by which high C_ant_ concentrations (>45.0 μmol kg^−1^) reach down to ~400 dbar (1^st^ deepening stage). The air–sea heat fluxes and mixing processes along the northward progression of the C_ant_-loaded NACW lead to a densification of NACW and its transformation into SPMW as it reaches the Iceland Basin. This transformation causes a deepening of C_ant_ down to ~600 dbar in this region (2^nd^ deepening stage). The average C_ant_ concentration within this newly formed SPMW (57.2 ± 5.8 μmol kg^−1^ for *σ*_θ_ < 27.52 kg m^−3^) is lower than upstream due to the C_ant_ content being spread out in the vertical. Further west, the Reykjanes Ridge is identified as a key topographic feature that marks a steep transition between the Iceland Basin and the Irminger Basin with markedly different vertical C_ant_ distributions. In the Irminger Sea, the C_ant_ signal (>35.0 μmol kg^−1^) deepens until 1400 dbar (3^rd^ deepening stage) and is constrained within the LSW layer, highlighting the role of the LSW formation on the C_ant_ deepening. The deepening is even more pronounced in the Labrador Sea, where the C_ant_ signal, still confined within the LSW, reaches 1800 dbar (4^th^ deepening stage). The newly formed LSW joins the Deep Western Boundary Current as part of the North Atlantic Deep Water (NADW), spreading the C_ant_ footprint downstream towards the ocean interior. Further evidence of this C_ant_ trapping within the LSW and its propagation downstream is provided by our data east of the Flemish Cap transition zone, where a relative C_ant_ maximum can be identified within the LSW layer underneath the NACW (Fig. 2b). As surface waters spread through the different basins of the SPNA, we also inferred water masses that have favorable conditions to uptake additional atmospheric C_ant_, by means of the C_ant,def_ proxy. Our results indicate that air-sea C_ant_ fluxes are likely to occur all over the region, and as far west as in the Labrador Basin.

Deep water formation is a broadly known and acknowledged mechanism for C_ant_ penetration in the interior of the North Atlantic Ocean^2,6,7^. Relying on Argo-O_2_ data and existing methods, we presented an original approach that allowed us to describe this C_ant_ deepening at the finest spatiotemporal scale to date. Our results revealed the role of the SPMW formation as an early stage of subpolar C_ant_ deepening. This result reinforces the idea that C_ant_ deepening occurs throughout the whole SPNA gyre and is not only linked to the main convective sites (i.e., Irminger and Labrador Basins), as traditionally attributed^7,16^. Mainly uptaken from the atmosphere in the subtropics^2^, C_ant_ is transported northwards towards the SPNA region by the NAC^6^, which at these latitudes is the main component of the upper AMOC limb. The quasi-lagrangian Argo-float trajectories are a suitable and timely means for describing this horizontal C_ant_ journey. Despite lateral advection by the upper AMOC limb, being the main source of C_ant_ in the region, the contribution from ocean C_ant_ uptake is also relevant^6,12^. By means of the C_ant,def_ proxy, the Argo-O_2_ data also support the hypothesis of surface air-sea C_ant_ enrichment all along the subpolar gyre domain by an outcropping of the low-carbon nutrient stream isopycnal range^12^. Given the dominant role of lateral advection in C_ant_ distribution, it is not surprising that AMOC variability affects the northward C_ant_ transport towards the SPNA and its storage rates^6,11^. Under current climate change, the AMOC is projected to weaken^37^, and we could thus expect a concomitant decrease in C_ant_ storage rates and content in the SPNA. Nevertheless, the northward oceanic C_ant_ transport might still be subject to continuous increase in response to the rise in anthropogenic CO_2_ emissions^11,38^. Estimates on a finer spatio-temporal scale, such as those based on Argo-O_2_ observations, should be sustained in the long term to determine which effect will be dominant in the future.

Our case study has shown that neural networks combined with high-quality in situ Argo-O_2_ measurements and a back-calculation method can effectively be used to retrieve C_ant_ concentration through the three-dimensional estimates of oceanic variables (nutrients, DIC, total alkalinity). These oceanic variables are needed to compute C_ant_ at a higher spatio-temporal resolution than that available from the current biogeochemical (BGC) observation networks. Hence, the methodology presented here, enchaining Argo-O_2_ observations and existing methods, opens up a promising step forward to study C_ant_ distribution, its variability, and the processes driving these variations, which are crucial challenges regarding the ocean carbon sink^39^. The global nature of the Argo-O_2_ array gives the opportunity to significantly increase the spatio-temporal coverage of current C_ant_ estimates. However, we can only guarantee the validity of our approach in the case-study region and for the period (1970–2020) over which the ship-based data used for training and validation of the neural networks were collected (GLODAPv2.2020^40^). Neural networks can only reproduce what they have learned. Therefore they must be trained with new data as the ocean changes, which is particularly true for the SPNA gyre where interannual to decadal changes are considerable (e.g., cold blob^41^ or great salinity anomaly^42^). The importance of high-accuracy in situ data measurements for training the neural networks cannot be disregarded, as such, the continuation of ship-based observations should be assured. Another important consideration of our results and their interpretation lies within the averaged total uncertainty of our method, which is ±5.9 µmol kg^−1^. Any spatio-temporal change in C_ant_ concentration below two times this value should be interpreted with caution. This current methodological uncertainty could possibly be reduced by using data from the growing BGC-Argo database^43,44^ to train the neural networks. Moreover, BGC-Argo floats, equipped with pH and nitrate sensors, could be directly used to derive C_ant_, hence providing independent validation C_ant_ estimates when both BGC-Argo and Argo-O_2_ floats are concomitant in space–time. However, BGC-Argo floats were not included in our study because available floats did not match our selection criteria. In view of the above, there is, therefore, a complementarity between the scarce high-quality ship-based BGC measurements and the high spatiotemporal BGC-Argo and Argo-O_2_ observations. This complementarity is even more marked because Argo data need to be adjusted (QCed) against ship-based data. The Argo-O_2_ float data presented in this case study reached a maximum pressure level of 2000 dbar, limiting our result to the top 2000 dbar of the water column. The OneArgo mission calls for the deployment of 1250 floats capable of reaching 4000 or 6000 dbar^45^. Using Deep-Argo floats equipped with dissolved oxygen sensors could thus strengthen our findings and improve our knowledge about the mechanisms controlling the deep/abyssal C_ant_ transport. For instance, the role of the Iceland-Scotland Overflow Water (ISOW), flowing below 2000 dbar, could not be identified here even though this water mass is known to contain C_ant_^11^.

Finally, it is worth mentioning that Earth system models tend to misrepresent the vertical penetration of C_ant_ due to the shallow and weak formation of NADW^46^, even if this issue is better addressed in the latest generation of CMIP6 models^18,47,48^. The results of this study provide insights subject to improving the representation of the key pathways for the deep C_ant_ propagation in Earth system models. Implementing a better representation of these pathways would definitely ameliorate the simulated vertical distribution of C_ant_ and, ultimately, improve climate predictions.

## Methods

### Argo float observations

To highlight the C_ant_ penetration into the deep ocean, we use Argo-O_2_ floats, giving the opportunity to obtain one C_ant_ profile every 10 days. We selected the Argo-O_2_ floats that (1) followed the cyclonic pathway of the SPNA gyre, (2) had a lifetime longer than 3 years, and (3) crossed the A25 OVIDE hydrographic section^49^, which will serve as a reference dataset for validation. Three floats matched these criteria, comprising a total of 651 profiles of pressure (P), temperature (T), salinity (S), and oxygen (O_2_)^50^ within the region 47–65°N; 15–65°W (Supplementary Fig. 1). Data span from July 2012 to April 2018. Only data adjusted in delayed mode with a quality flag of 1 or 2 (good or probably good data)^51^ were used in our analysis (Supplementary Table 1). The accuracy of the data is assumed to be 0.002 °C, 0.01, and 2.4 dbar for temperature, salinity, and pressure, respectively^52^, and better than 3 μmol kg^−1^ for oxygen^53^.

### Estimating biogeochemical variables with neural networks

To derive the biogeochemical variables (nutrients, DIC, and A_T_) needed to compute C_ant_, we rely on predictive neural networks. Neural networks (NN) are machine learning algorithms based on a multi-layer perceptron^54,55^, that are trained and validated against observations. The input data of the neural networks are, in this study, Argo P/T/S/O_2_ measurements. We used two neural networks, namely, ESPER_NN^25^ and CANYON-B, associated with its routine CONTENT^26^. ESPER_NN was used to obtain the macronutrients (phosphate, nitrate, and silicate) using P, T, S, O_2_, location, and time as predictors. ESPER_NN was adopted over other ESPER methods (i.e., ESPER_LIR and ESPER_Mixed) because it gives the lowest biases and root mean square errors over the global ocean for the predicted macronutrients^25^. ESPER_NN reproduces the validation dataset with average biases and errors of ~2% for macronutrients (Supplementary Table 1)^25^. However, in the North Atlantic Ocean, ESPER_NN gives uncertainties of ~1.3% for the predicted carbonate variables (A_T_ and DIC), which is higher than the previous NN. Consequently, A_T_ and DIC were computed with CANYON-B^26^, and the outputs of CANYON-B were passed through the CONTENT routine. This routine ensures consistency between carbonate variables and thus reduces the uncertainties of the carbonate system variables to ~0.5% for A_T_ and DIC (Supplementary Table 1). We used neural networks rather than climatological products (such as, e.g., GLODAPv2 climatology^56^) because gridded climatologies are unable to capture changes in carbon variables in regions where water masses move laterally due to mesoscale processes or rapid circulation changes. This is because climatologies represent the mean field in which the variability has been smoothed out. In contrast, neural networks, such as ESPER_NN and CANYON-B, derive biogeochemical variables based on water mass characteristics, hence they can cope with such changes. In particular, the use of Argo-O_2_ float data as input to the neural networks reinforces this water-mass change tracking capability due to the quasi-lagrangian behavior of the Argo-O_2_ floats and their temporal resolution, three times greater than monthly climatological products.

### Anthropogenic carbon estimates

To determine the anthropogenic carbon fraction (C_ant_) from DIC, we used the carbon-based back-calculation φC_T_^O^ method^17,27^. This method has been widely applied to study the inventory of C_ant_, its storage rates, its variability^14,17,57^, and the influence of C_ant_ on ocean acidification^58^. In any back-calculation method, C_ant_ is computed as a two-step approach: (1) the changes in DIC due to biological activity occurring since a water parcel has left the ocean surface are removed, and (2) the pre-industrial preformed DIC is also removed, with the residual being interpreted as C_ant_. The φC_T_^O^ method (Supplementary Equation 1) presents two main advantages compared to other back-calculation methods. First, this method accounts for the spatiotemporal variability of the preformed A_T_. Second, the parameterization of the preformed A_T_ and disequilibrium terms are determined using the subsurface layer as a reference^27^. These two modifications improve the C_ant_ estimates in cold and deep water formation regions subject to strong mixing processes, such as the SPNA gyre. A study comparing observational methods to estimate C_ant_ in the Atlantic Ocean, including the TTD^9^, the TrOCA^59^, the C^o^_IPSL_^60^, the ΔC*^61^, and the φC_T_^O^ method^17^, demonstrated that the latter provided the closest value to the average of all methods for the whole latitudinal range^27^. Based on that, we selected the φC_T_^O^ method to perform our analysis. The input variables for this method are date, geographical location, T, S, O_2_ (in this study, the Argo variables), the macronutrients (obtained from ESPER_NN), plus A_T_ and DIC (computed from CANYON-B and CONTENT). The φC_T_^O^ method requires date as input because it adjusts the oceanic C_ant_ concentration against the corresponding atmospheric *p*CO_2_. It also requires the geographical location (latitude and longitude) to define the water masses involved in the computation of the preformed A_T_ and disequilibrium terms. All the input variables have associated uncertainties that propagate through C_ant_ calculations. The uncertainties on Argo pressure, temperature, and salinity data are 2.4 dbar, 0.002 °C, and 0.01, respectively^52^. These uncertainties relate to the instrument’s performance. The uncertainties on O_2_ data are about 3.5 μmol kg^−1^ (Supplementary Table 1). The uncertainties of the predicted variables A_T_, DIC, and macronutrients are, on average, 10.85, 10.48, and 0.73 μmol kg^−1^, respectively (Supplementary Table 1). These NNs uncertainties are provided as part of the NNs predictive outputs and they result from the combination of training measurement uncertainties, the Bayesian method uncertainties, and the NNs input sensitivities^26^. Taking all of them into account, we quantified the uncertainty associated with our C_ant_ estimate by randomly generating 100 C_ant_ fields (Supplementary Table 1) using a Monte Carlo method^62^. The standard deviation from these C_ant_ fields fluctuates between ±5.4 μmol kg^−1^ and ±10.2 μmol kg^−1^ (Supplementary Fig. 2) and the overall average value (for the three floats) is ±5.9 μmol kg^−1^. It is worth highlighting that the uncertainties of the modeled tracer fields (Supplementary Table 1) are evidently greater than the corresponding in situ measured variables (i.e., by 1.5 μmol kg^−1^ for oxygen). However, the uncertainties of the predicted variables are yet reasonably small to serve as inputs to the back-calculation method. Consequently, they allow an estimation of C_ant_ that is statistically significant (and whose final uncertainties lie within 9% to 19% of the signal). To further validate our approach, we compared these ArgoTSO_2_-NN-based C_ant_ estimates with ship-based C_ant_ estimates obtained during OVIDE cruises^49,63^. We identified Argo-O_2_ profiles within the 35 km radius and less than 7 month-timespan of an OVIDE CTD cast (Supplementary Fig. 2). For each CTD cruise cast, C_ant_ is estimated from in situ bottle-sample measurements of nutrients, oxygen, A_T_ and pH, via the φC_T_^O^ method (standard procedure^11^). Emulating the approach proposed in this study for the Argo-O_2_ floats (TSO_2_-NN procedure), we re-estimated C_ant_ by using the ship-based P/T/S/O_2_ data as input to the neural networks to obtain nutrients, DIC, and A_T_, and then applied the φC_T_^O^ method. These C_ant_ profiles (ship-based standard and ship-based TSO_2_-NN) were then compared to our Argo-based C_ant_ estimates (Argo-based TSO_2_-NN procedure), and all of them agreed well within the averaged method uncertainty of ±5.9 μmol kg^−1^ (Supplementary Fig. 2). This comparison indicates that the C_ant_ values obtained from ship-based measurements and C_ant_ estimates calculated via Argo-O_2_ data and neural networks give consistent results.

We also computed a proxy that allows the indirect assessment of the air–sea C_ant_ uptake capacity, the C_ant_ deficit (C_ant,def_, Supplementary Fig. 3). This proxy was defined by Ridge & McKinley^12^ as the difference between the C_ant_ concentration of a water parcel and a reference (${\langle {{{{{\rm{C}}}}}}_{{{{{\rm{ant}}}}}}\rangle }_{{\sigma }_{\theta } < 26.5}$) computed as the average C_ant_ concentration between the surface and the potential density (*σ*_θ_) of 26.5 kg m^−3^. This isopycnal delimits the subtropical surface waters assumed to be in equilibrium with the current atmosphere. A water parcel with a negative C_ant,def_ value indicates that this parcel has a deficit of C_ant_. If transported to the surface, this water parcel will have a lower *p*CO_2_ compared to a water parcel without this deficit, hence favoring the air–sea C_ant_ uptake. Usually, C_ant,def_ becomes more and more negative with depth (Supplementary Fig. 3), meaning that the deep waters would uptake a higher amount of C_ant_ compared to the subsurface waters if they were transported to the ocean surface. The reference value is computed for 2002 with the GLODAPv2 dataset^56^, considering the averaged C_ant_ concentration in surface waters (*σ*_θ_ < 26.5 kg m^−3^) offshore the Venezuelan coasts (10–20°N; 70–64°W), and is ${\langle {{{{{\rm{C}}}}}}_{{{{{\rm{ant}}}}}}\rangle }_{{\sigma }_{\theta } < 26.5}^{2002}\,$ = 51.5 μmol kg^−1^. We assume that our reference water mass offshore the Venezuelan coasts takes 5 years to arrive in the Iceland and Irminger basins^64^. For a given float, we use that reference value rescaled in time to 5 years before the time of its first profile, using the exponential equation of Carter et al.^25^. Assuming that due to its lagrangian behavior, the float follows the transformation of the same water mass, we kept the same reference value along the float trajectory within the subpolar gyre. However, this assumption is not valid for float 5904988 when it drifted from Region 4 to Region 1 and entered the North Atlantic Current, where we re-initialized the reference value.

### Mixed layer depth and water mass definition

To determine the mixed layer depth (MLD) along the Argo float trajectories, we use the threshold method, which is based on a density difference of 0.01 kg m^−3^ ^65^ between the surface and the base of the mixed layer^66^. Since this method tends to overestimate the MLD during deep convection events in winter^67^, we conducted a visual inspection^68^ to verify the MLD values during these events. When the MLD values disagreed between the threshold method and the visual inspection, we kept the value determined by visual inspection.

The North Atlantic is composed of several water masses (Fig. 1), most of which can be identified by their potential temperature (*θ*), *S*, *σ*_θ_, O_2_, and potential vorticity. Here, the potential vorticity is calculated via the Brunt–Väisälä frequency, the gravity, and the Coriolis parameter. In the upper layers, the North Atlantic Current (NAC) flowing from the subtropics to the subpolar regions (Fig. 1), carries North Atlantic Central Water (NACW)^30^. The lower limit of this water mass is defined by *σ*_θ_ = 27.2 kg m^−3^ ^69^ (Supplementary Fig. 4). Continuous air-sea interaction and winter convection along the NAC path cools and freshens the NACW, leading to its transformation into Subpolar Mode Waters (SPMWs)^31,32^. SPMWs are characterized by nearly homogeneous properties, with *S* > 34.98, *σ*_θ_ < 27.71 kg/m^3^, and a potential vorticity lower than 6 × 10^−11^ m^−1^ s^−1^ ^31,70^ (Figs. 3 and 4). At intermediate depths (400–2000 dbar), in the subpolar gyre, the Labrador Sea Water (LSW) is located between 27.71 < *σ*_θ_ < 27.80 kg/m^3^. The LSW is formed by deep winter convection in the Labrador Sea^71^ and Irminger Sea^36,65^, trapping large amounts of O_2_ and C_ant_ during its formation^72^. In those basins, we defined the LSW by O_2_ ≥ 290 μmol kg^−1^ ^34^. However, along its path, the LSW loses O_2_ due to mixing and biological activity. Therefore the O_2_ criteria is not fully reliable to identify the LSW core away from its source regions. As a consequence, outside the Irminger and Labrador Seas we identify the LSW by S < 34.94^53^. At deeper levels (up to 2000 dbar), the Iceland-Scotland Overflow Water (ISOW) flows along the Reykjanes Ridge and is identified by *σ*_θ_ > 27.80 kg m^−3^ and S > 34.94^34^ (Supplementary Fig. 4).

### Eddy identification

To detect the eddies along the Argo-float pathways, we run the autonomous eddy identification scheme of Faghmous et al.^73^, which monitors mesoscale ocean eddy activity in global sea level anomaly (SLA) dataset (http://www.aviso.altimetry.fr/duacs/). The scheme starts from the simple notion that every eddy has a single extremum, defined as a grid cell whose SLA is higher or lower than its 24 neighbors in a 5 × 5 grid. To determine the contour of the eddy, the algorithms search for the largest possible contour that would allow the feature not to violate the assumption that an eddy can have only a single extremum within its interior. For each eddy feature identified at time *t*, the eddies at time *t* + 1 are searched to find the closest feature within a pre-defined search space. When a feature at time t is associated with another feature at time *t* + 1, their sizes are compared to ensure that they are reasonably similar from a physical point of view. There are two major uncertainties associated with this eddy identification method. First, the use of a geometric eddy definition, as opposed to a physical one, introduces uncertainties in the eddy boundaries because they are not necessarily associated with the physical properties of the eddy. Second, as the method constrains features to have a single extremum, small features that are in close proximity might cancel each other out and not be detected. Despite these limitations, the success discovery rate of the autonomous eddy identification algorithm is 96.4%^73^.

### Reporting summary

Further information on research design is available in the Nature Portfolio Reporting Summary linked to this article.

### Supplementary information

Supplementary Information

Peer Review File

Reporting Summary

## Data Availability

The original Argo data can be freely downloaded on the Euro Argo Data Selection platform (https://dataselection.euro-argo.eu/). Our C_ant_ estimates, needed to evaluate the conclusion of the paper, can be downloaded on Zenodo (10.5281/zenodo.7071614).

## Code availability

The Matlab codes used for the simulation are available from the corresponding author upon request.
