# Supplementary material for: Anthropogenic carbon pathways towards the North Atlantic interior revealed by Argo-O2, neural networks and back-calculations
Source: Nat Commun. 2024 Feb 22;15:1630. doi: 10.1038/s41467-024-46074-5 (PMC10884407; doi:10.1038/s41467-024-46074-5)
Supplement: Supplementary file 1 — Supplementary Information [file 41467_2024_46074_MOESM1_ESM.pdf]

# Anthropogenic carbon pathways towards the North Atlantic interior revealed by Argo-$O_2$, neural networks and back-calculations

Rémy Asselot[1*], Lidia I. Carracedo[1], Virginie Thierry[1], Herlé Mercier[1], Raphaël Bajon[1] and Fiz F. Pérez[2]

[1]University of Brest, Ifremer, CNRS, IRD, Laboratoire d'Océanographie Physique et Spatiale (LOPS), F-29280, Plouzané, France.
[2]Instituto de Investigaciones Marinas (IIM, CSIC), calle Eduardo Cabello, 6, 36208, Vigo, Spain.

*Corresponding author. E-mail: remy.asselot@ifremer.fr

This file contains all the Supplementary Figures.
It also includes the main equation of the $C_{ant}$ computations, it describes the scaling method and the method used to determine the $C_{ant}$ uncertainties.

Supplementary Fig. 1: Geographical location of the three Argo-$O_2$ floats.
Supplementary Fig. 2: Comparison of $C_{ant}$ profiles between OVIDE, GLODAPv2 and Argo-$O_2$ data.
Supplementary Fig. 3: Section of $C_{ant}$ deficit (µmol kg$^{-1}$) along the water column.
Supplementary Fig. 4: Potential temperature - salinity diagrams with $C_{ant}$ displayed in color along isopycnals (kg m$^{-3}$).
Supplementary Fig. 5: Argo float 5904988.
Supplementary Fig. 6: Argo float 6901023.
Supplementary Fig. 7: Argo float 6901026.
Supplementary Fig. 8: Eddy locations and their past and future tracks.

Supplementary Table 1: Uncertainties on the input variables to calculate $C_{ant}$ for the 3 Argo floats.

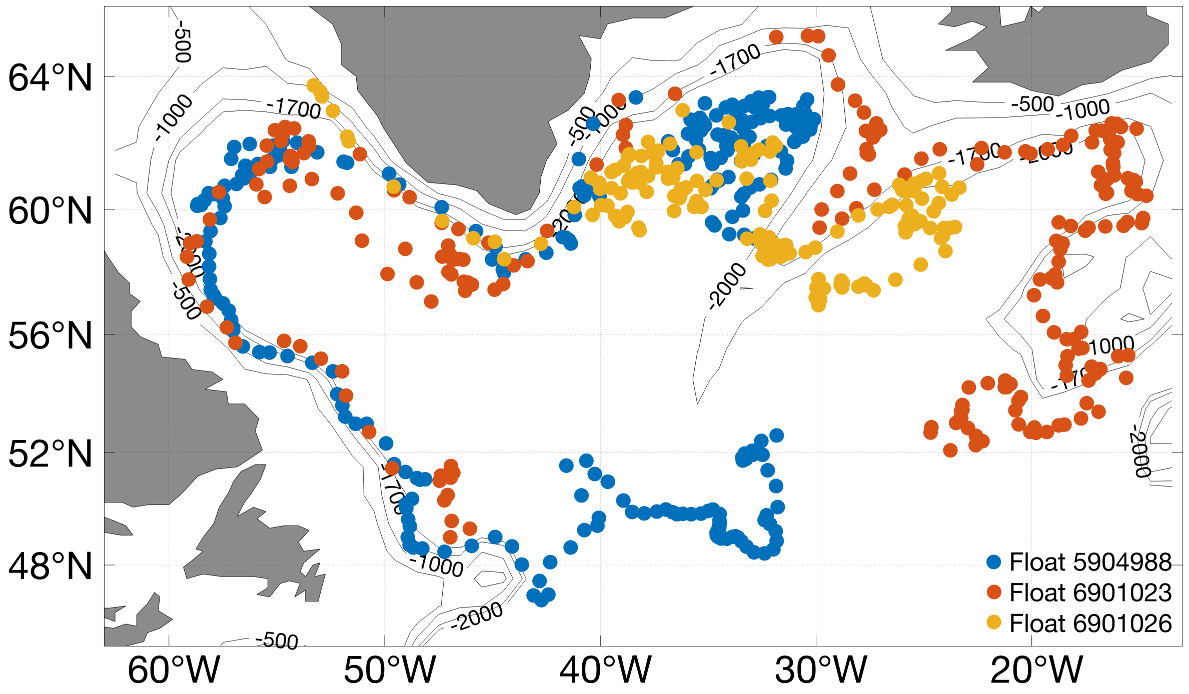


**Supplementary Fig. 1: Geographical location of the three Argo-$O_2$ floats.** Blue dots represent the location of the float 5904988. Orange dots depict the location of the float 6901023. Yellow dots represent the location of the float 6901026.

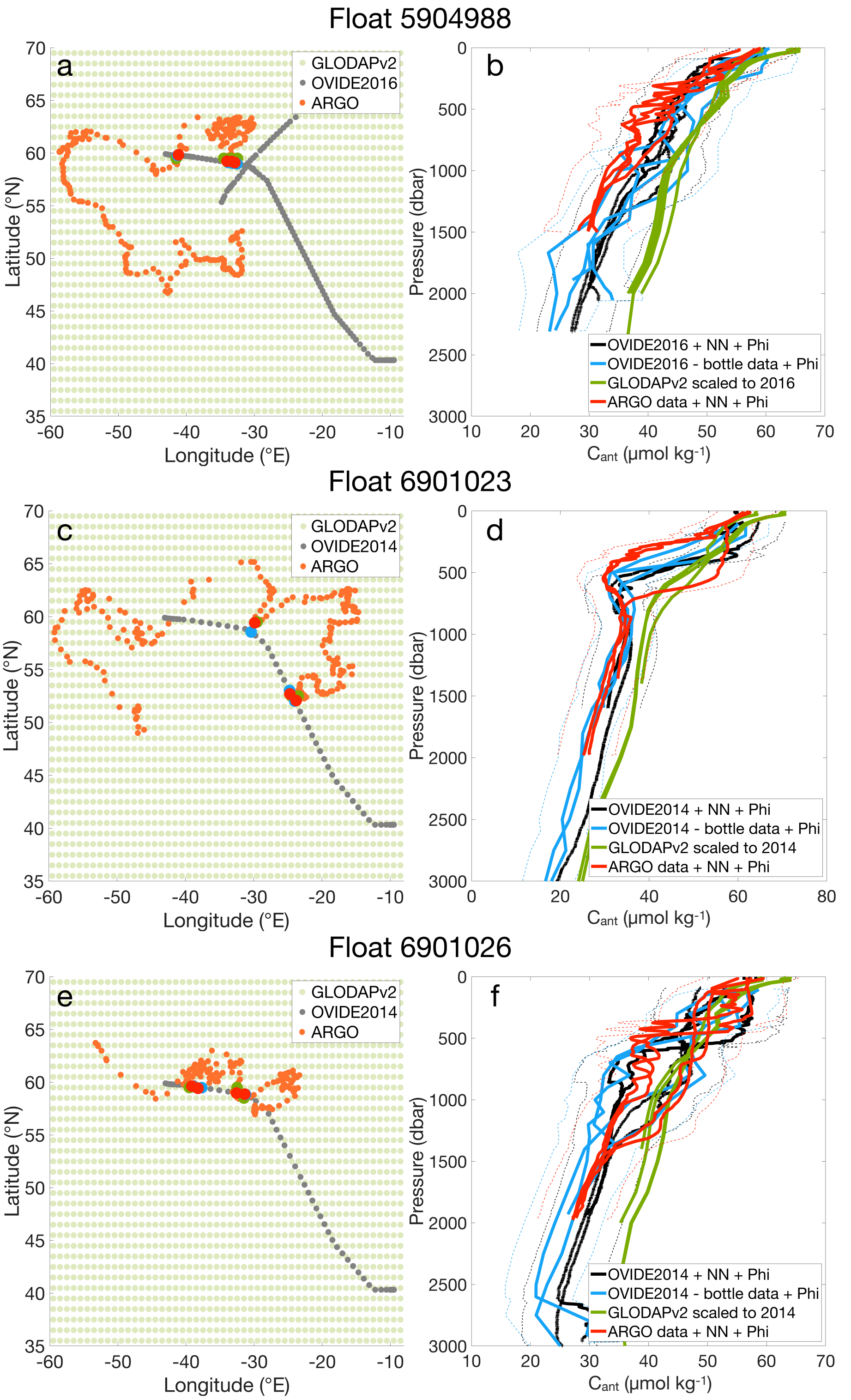


**Supplementary Fig. 2: Comparison of $C_{ant}$ profiles between OVIDE, GLODAPv2 and Argo-$O_2$ data.** Panels on the left show the location of the OVIDE (gray dots), GLODAPv2 (light green dots) and Argo-$O_2$ (orange dots) profiles for **a**, Float 5904988. **c**, Float 6901023. **e**, Float 6901026. The red, blue and dark green dots indicate the location of the $C_{ant}$ profiles compared on the right panels. Panels on the right show the $C_{ant}$ profiles of the Argo-$O_2$ data (Argo-based $TSO_2$-NN procedure, solid red), OVIDE bottle data (ship-based standard procedure, solid blue), OVIDE gridded product (ship-based $TSO_2$-NN procedure, solid black) and GLODAPv2 (solid green) for **b**, Float 5904988. **d**, Float 6901023. **f**, Float 6901026. The dashed red, blue and black lines represent the methodological uncertainties of different methods to compute $C_{ant}$.

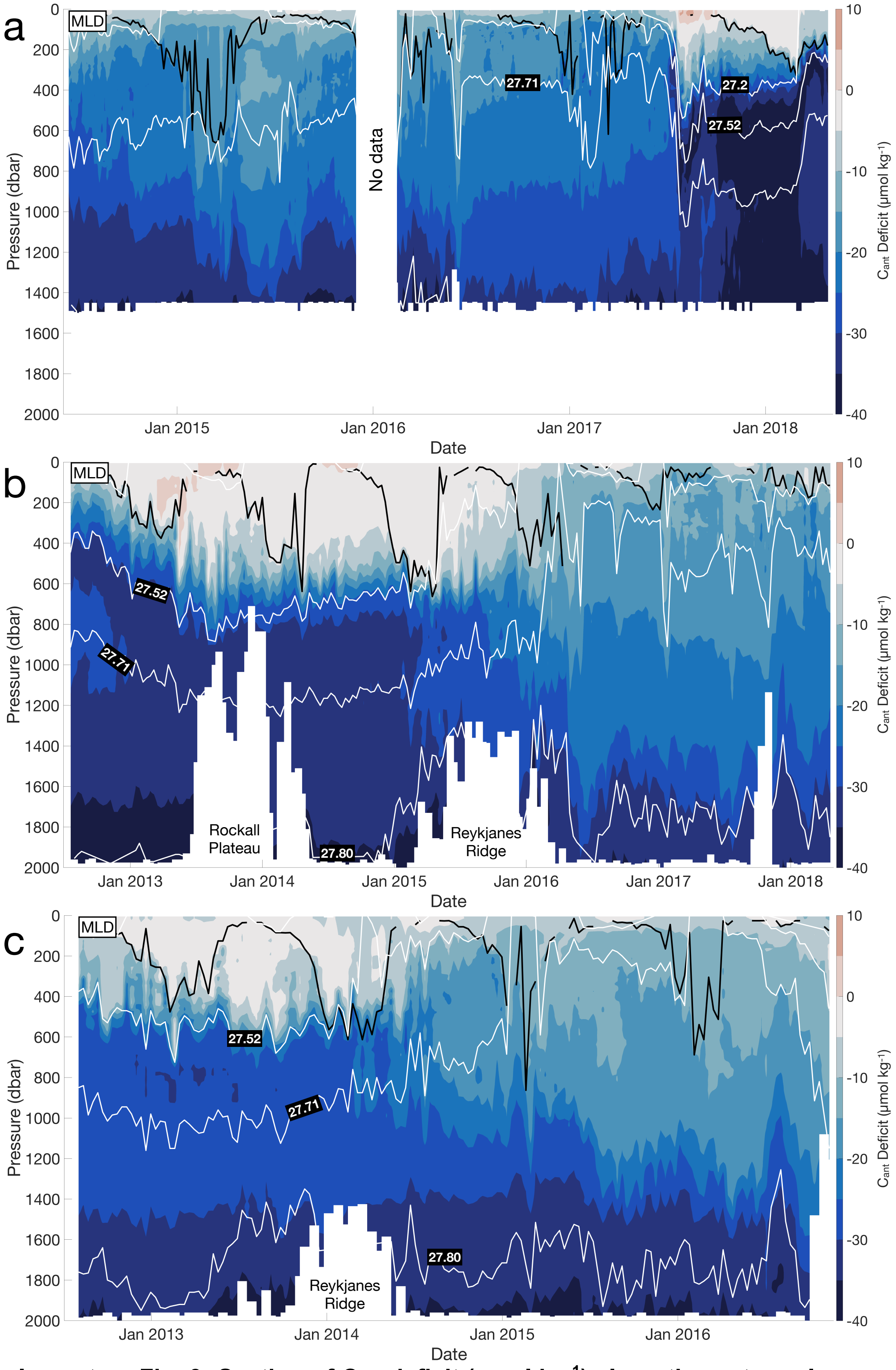


**Supplementary Fig. 3: Section of $C_{ant}$ deficit (μmol kg$^{-1}$) along the water column. a**, For float 5904988. **b**, For float 6901023. **c**, For float 6901026. A water parcel with a negative $C_{ant,def}$ value (blue color) indicates that this particular parcel has a deficit of $C_{ant}$ and is able to uptake $C_{ant}$ from the atmosphere. The black line represents the mixed layer depth. The white lines indicate the isopycnals (kg m$^{-3}$).

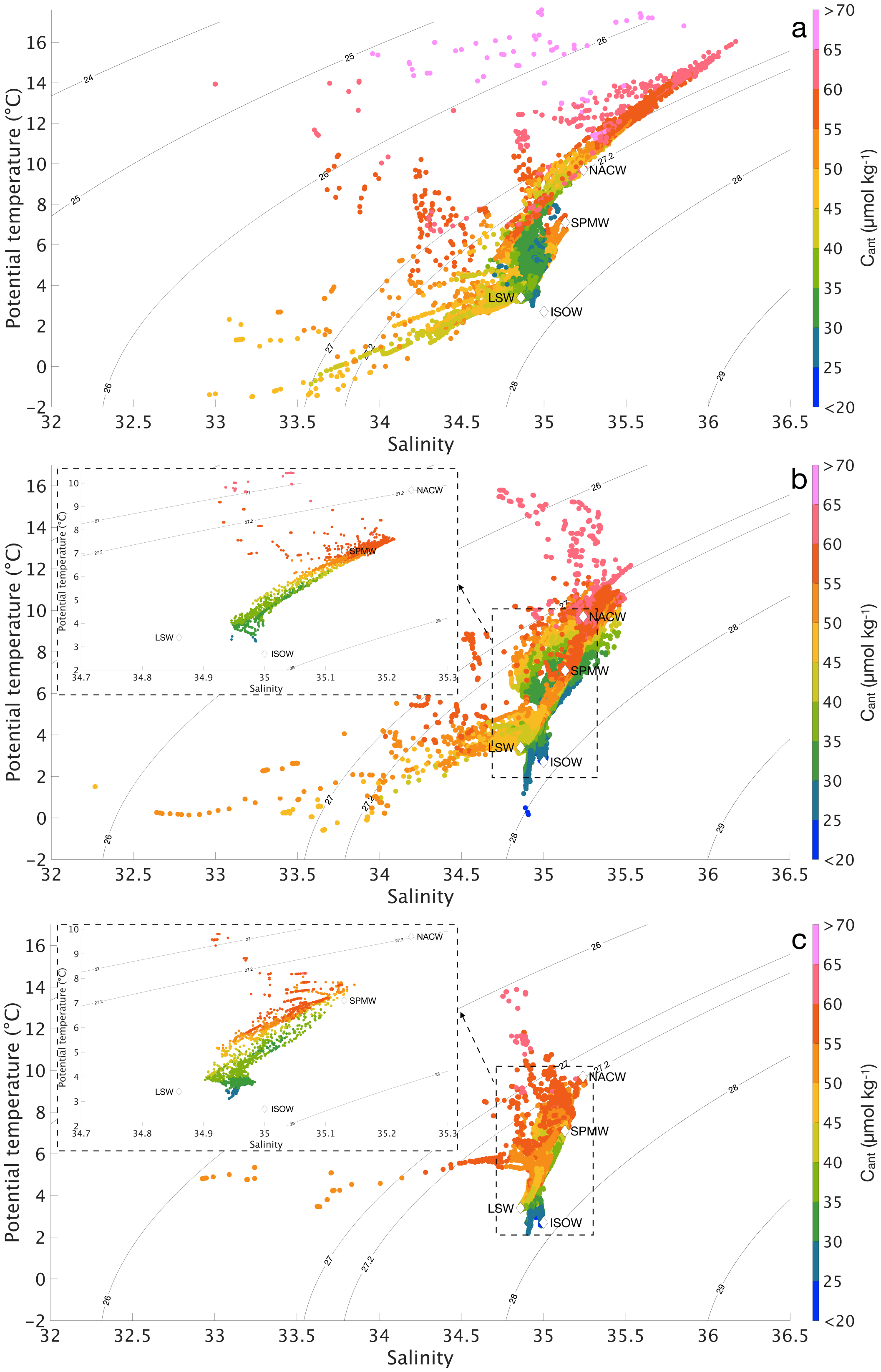


**Supplementary Fig. 4: Potential temperature - salinity diagrams with $C_{ant}$ displayed in color along isopycnals (kg m$^{-3}$). a**, Float 5904988. **b**, Float 6901023. **c**, Float 6901026. For the panels **b** and **c** the zooms represent the Argo profiles above the Reykjanes Ridge only. The source water types are defined following García-Ibáñez et al. (2016) and Talley et al. (2011). NACW = North Atlantic Central Water; SPMW = Subpolar Mode Water; LSW = Labrador Sea Water; and ISOW = Iceland–Scotland Overflow Water.

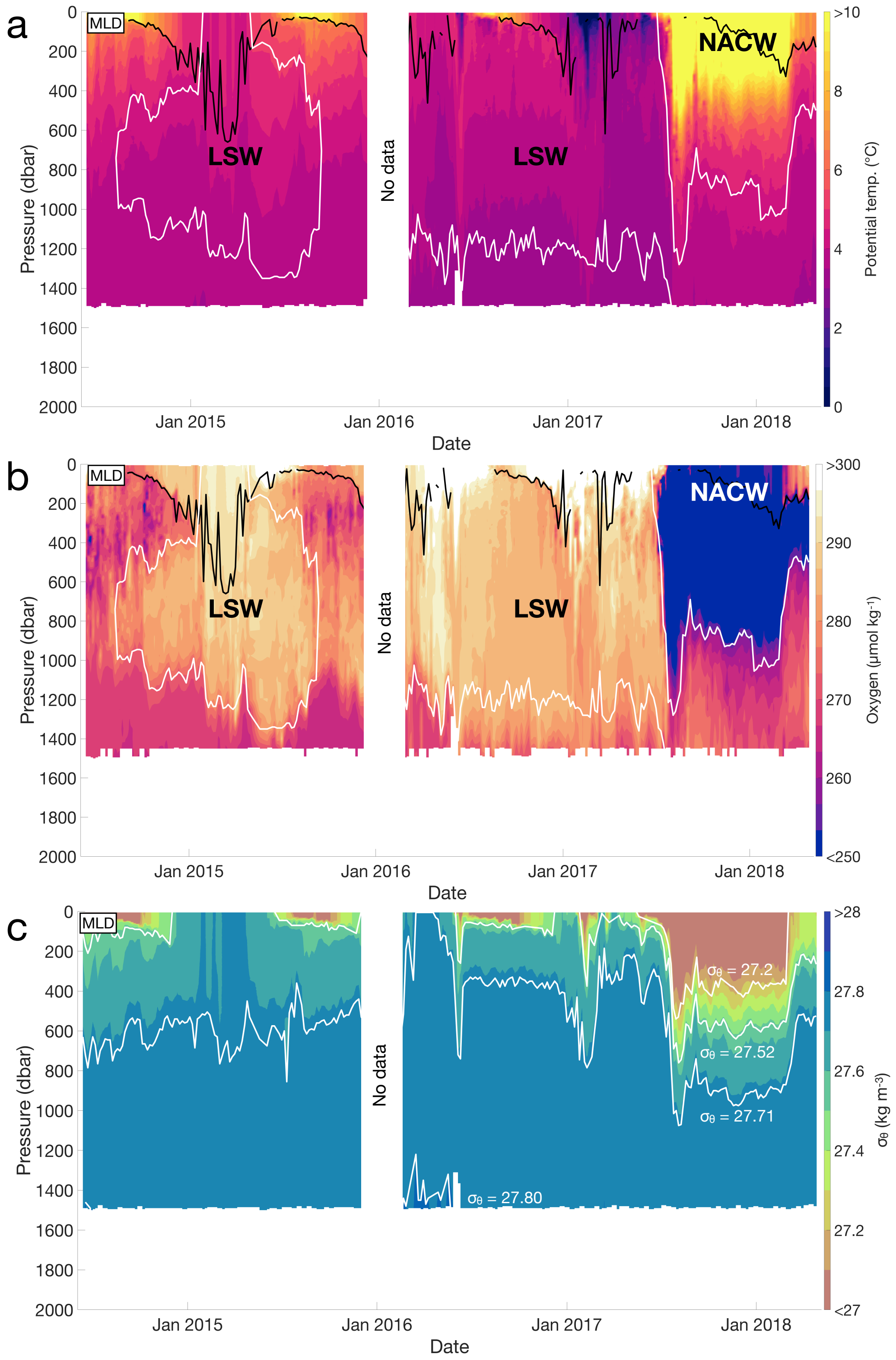


**Supplementary Fig. 5: Argo float 5904988. a**, Potential temperature (°C) along the water column. The black line represents the MLD. The white lines represent the limits of the Labrador Sea Water (LSW), defined by $O_2 \geq 290$ µmol kg$^{-1}$ in the Labrador and Irminger Seas and by S < 34.94 outside these two basins. **b**, Dissolved oxygen (µmol kg$^{-1}$) along the water column. **c**, Potential density (kg m$^{-3}$) along the water column. The white lines represent the isopycnals separating the water column in different layers.

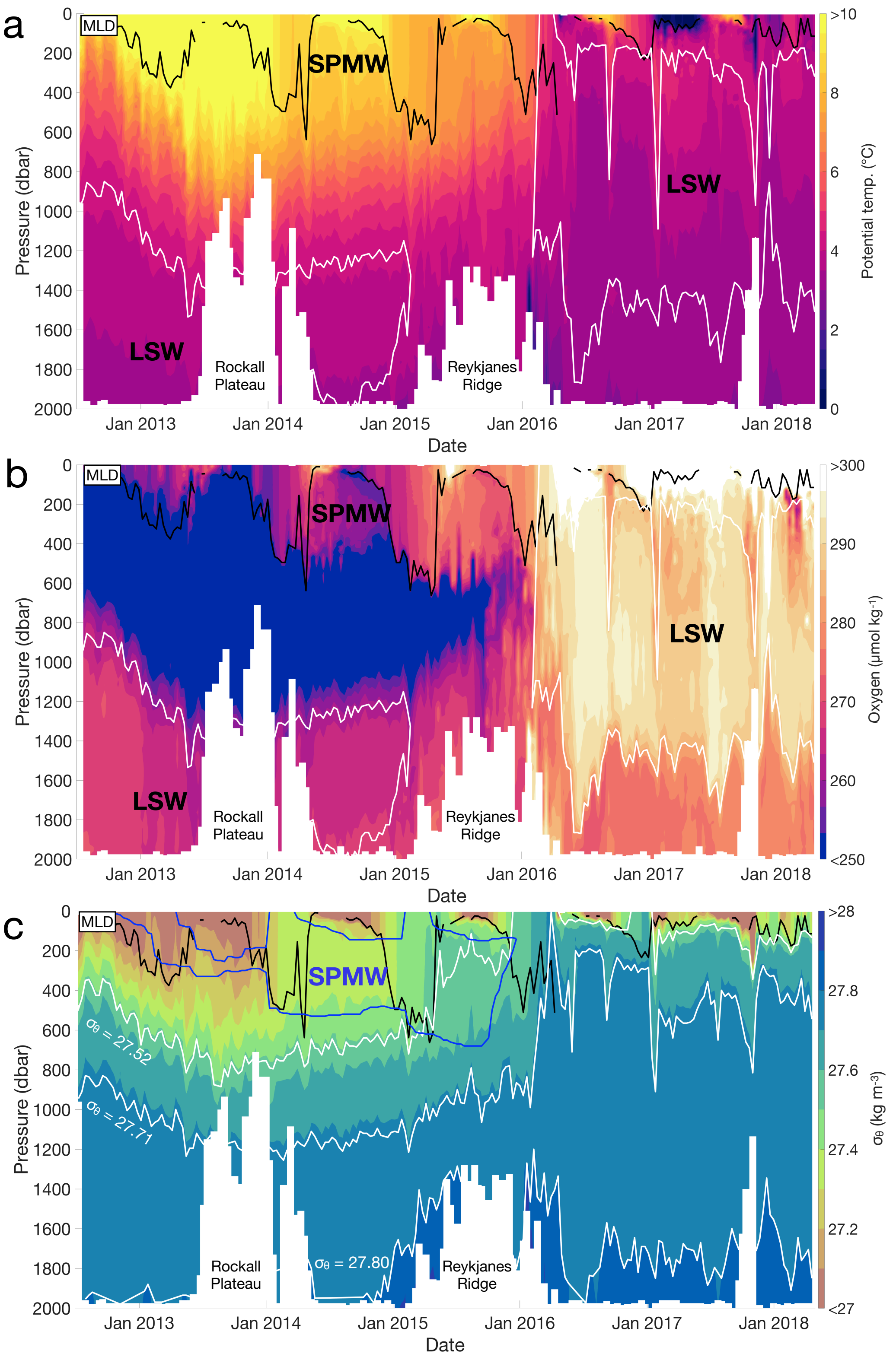


**Supplementary Fig. 6: Argo float 6901023. a**, Potential temperature (°C) along the water column. The black line represents the MLD. The white lines represent the limits of the Labrador Sea Water (LSW), defined by $O_2 \geq 290$ µmol kg$^{-1}$ in the Labrador and Irminger Seas and by S < 34.94 outside these two basins. **b**, Dissolved oxygen (µmol kg$^{-1}$) along the water column. **c**, Potential density (kg m$^{-3}$) along the water column. The white lines represent the isopycnals that separate the water column in different layers. The blue line represents the limits of the Subpolar Mode Waters, defined by a potential vorticity lower than 6x10$^{-11}$ m$^{-1}$s$^{-1}$.

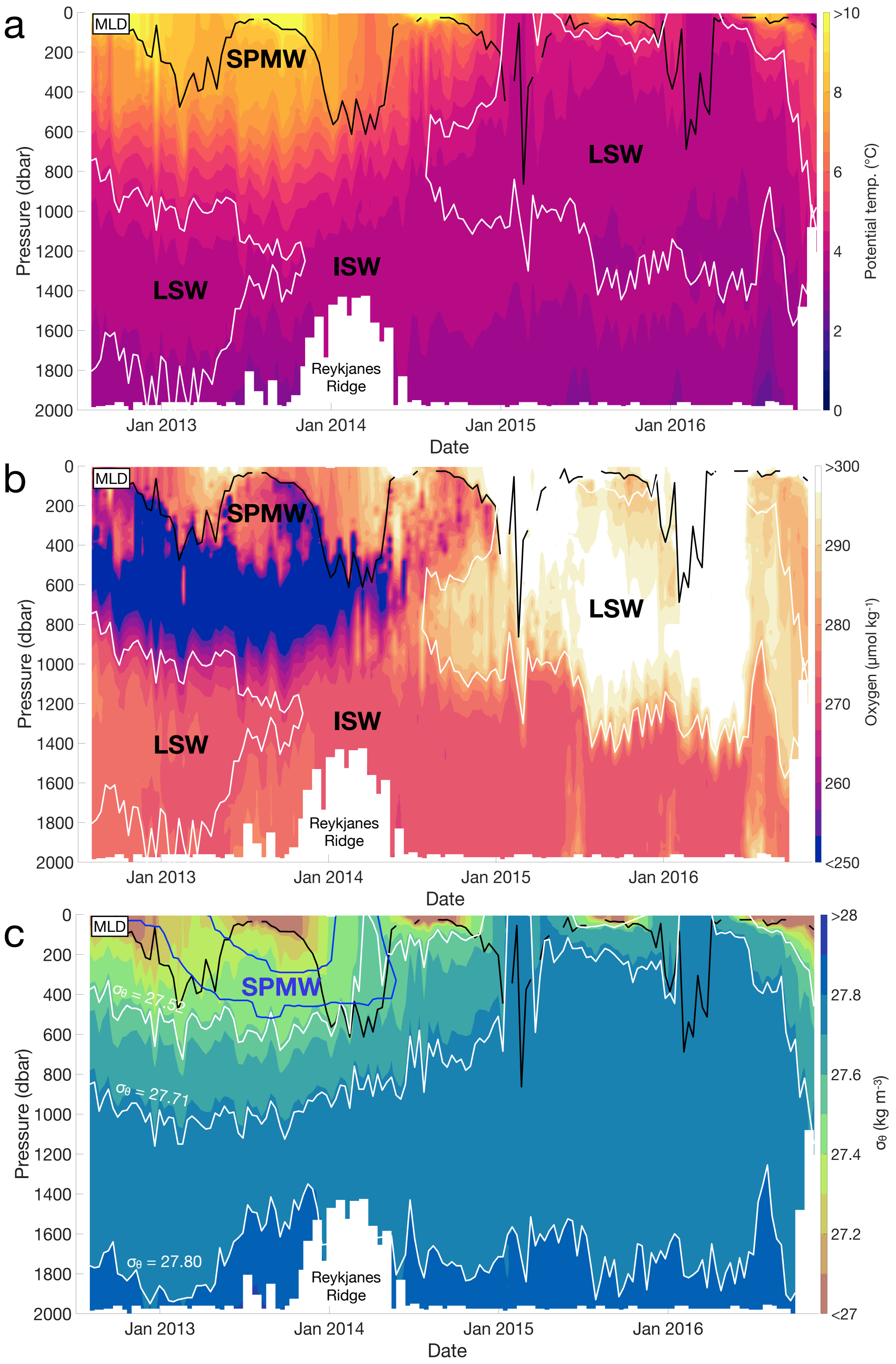


**Supplementary Fig. 7: Argo float 6901026. a**, Potential temperature (°C) along the water column. The black line represents the MLD. The white lines represent the limits of the Labrador Sea Water (LSW), defined by $O_2$ ≥ 290 µmol kg$^{-1}$ in the Labrador and Irminger Seas and by S < 34.94 outside these two basins. **b**, Dissolved oxygen (µmol kg$^{-1}$) along the water column. **c**, Potential density (kg m$^{-3}$) along the water column. The white lines represent the isopycnals that separate the water column in different layers. The blue line represents the limits of the Subpolar Mode Waters, defined by a potential vorticity lower than 6x10$^{-11}$ m$^{-1}$s$^{-1}$.

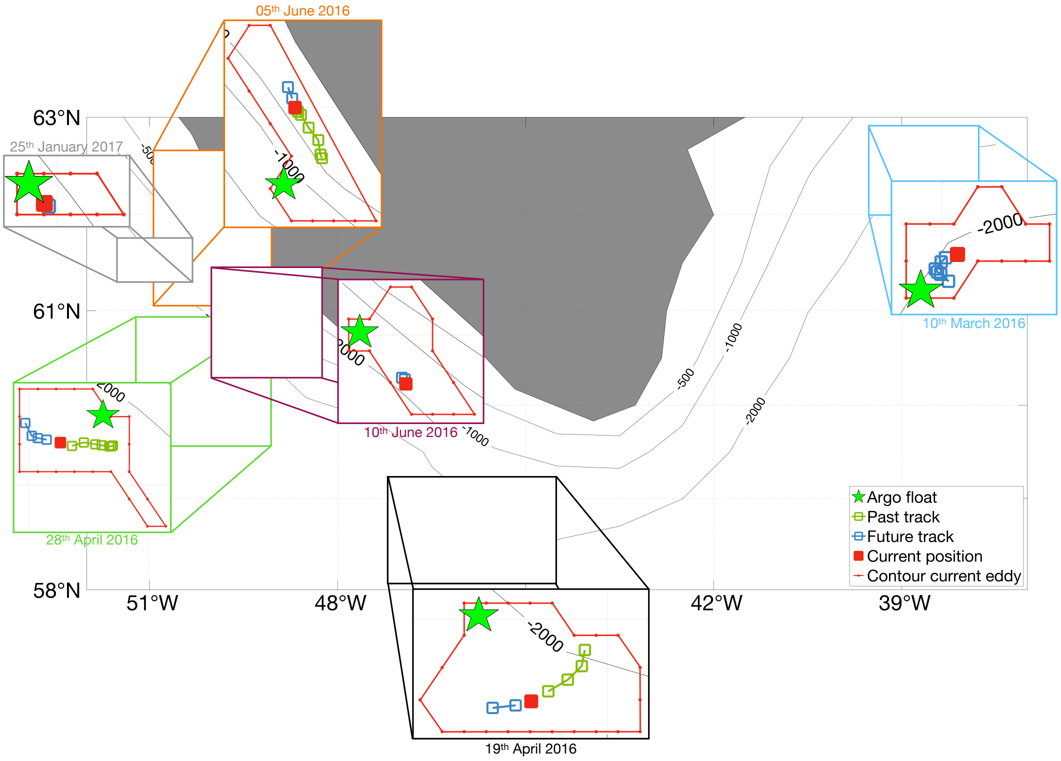


**Supplementary Fig. 8: Eddy locations and their past and future tracks.** The green stars in the orange and purple boxes represent the locations of the Argo float 5904988, also pointed out on Figure 2a. The green stars in the gray, green, black and blue boxes represent the locations of the float 6901023, also pointed out on Figure 3a. The identification of the eddies and their tracks follow the method of Faghmous et al. (2015).

## $C_{ant}$ computations

To compute $C_{ant}$ we rely on the $\varphi C_T^O$ method, which is a carbon-based method (Vazquez-Rodriguez et al., 2009). This method is an upgraded version of the ΔC* method (Gruber et al., 1996) and has been developed for the Atlantic Ocean. The $\varphi C_T^O$ method estimates $C_{ant}$ via the formula (Eq. 1) :

$$C_{ant} = \frac{\Delta C* - \Delta C_{dis}^{t}}{1 + \varphi\,|\Delta C_{dis}^{t}|\,/\,C_{ant}^{sat}} \qquad (1)$$

Where ΔC* is a quasi-conservative carbon tracer representing the uptake of $C_{ant}$ and the air-sea disequilibrium when a water parcel loses contact with the atmosphere (Gruber et al., 1996), $\Delta C_{dis}^{t}$ stands for the temporal variability of air-sea disequilibrium at time t, $C_{ant}^{sat}$ is the saturation of $C_{ant}$ which depends on the salinity and potential temperature and, finally, φ represents a constant term which depends on $\Delta C_{dis}$. In most of the cases, $C_{ant}$ obtained with the $\varphi C_T^O$ method will be lower than the ones obtained with the ΔC* method (Gruber et al., 1996) because the denominator of Eq. 1 is always higher or equal to one (Vazquez-Rodriguez et al., 2009). The $\varphi C_T^O$ method alone gives overall $C_{ant}$ uncertainties of ±5.2 µmol $kg^{-1}$.

## Scaling method

To scale the data, we followed Carter et al. (2021) who assumed that the exponential increase in atmospheric $C_{ant}$ results in an oceanic $C_{ant}$ increase rate proportional to atmospheric $C_{ant}$ concentrations. This assumption relies on the transient steady state hypothesis (Tanhua et al., 2007) which implies that the shape of a vertical $C_{ant}$ profile remains identical over time while $C_{ant}$ values increase exponentially (Eq. 2).

$$C_{ant}(t2) = C_{ant}(t1) \cdot exp(\alpha \cdot (t2 - t1)) \qquad (2)$$

Where $C_{ant}$(t2) stands for the $C_{ant}$ concentration (µmol $kg^{-1}$) at year t2, $C_{ant}$(t1) is the $C_{ant}$ concentration (µmol $kg^{-1}$) at year t1 and α is the scaling factor (no unit) which defines the change in the oceanic storage of $C_{ant}$ between two time points (t1 and t2). Originally, this factor is set to match the most recent global $C_{ant}$ distribution change of 28% (1.9% $yr^{-1}$) over the 1994-2007 period (Carter et al., 2021; Gruber et al., 2019). However, Gruber et al. (2019) indicate that the North Atlantic basin has a $C_{ant}$ distribution change 20% smaller than the global one. Consequently, for the 1994-2007 period, the $C_{ant}$ distribution change was 22.4% (1.52% $yr^{-1}$) for the North Atlantic and we used this value to scale α.

## Method uncertainties

The Argo floats selected for this study measure pressure (P), temperature (T), salinity (S) and oxygen ($O_2$). Based on data collected over the first 20 years of the Argo program and compared with independent observations from the GO-SHIP program, the accuracy of Argo data is 0.002°C for temperature, 2.4 dbar for pressure and 0.01 for salinity after delayed-mode adjustment (Wong et al., 2020). The best accuracy that can be reached for $O_2$ data with the present sensor type (oxygen optode) is 1-2 µmol

$kg^{-1}$, representing 0.5% of $O_2$ saturation (Grégoire et al., 2021). To obtain this accuracy, we need a cautious correction of the data to account for: (1) storage drift in $O_2$ sensitivity that occurs between calibration and deployment, representing a decrease in $O_2$ sensitivity of ~5% $yr^{-1}$ (Bittig et al., 2015); (2) *in situ* sensor drift that occurs during the multi-year deployment period (Bittig, Körtzinger, et al., 2018); (3) time response (Gordon et al., 2020) and (4) pressure dependent response of the sensor (Bittig, Körtzinger, et al., 2018; Racapé et al., 2019). The accuracy estimated after delayed mode correction (Thierry et al., 2021) can be higher than 2 µmol $kg^{-1}$ as each of those independent corrections cannot be systematically applied on the float data. They require some specificities, such as air measurement, timing of each observation or ship-based calibrated reference cast, that are not always available for all float types and all float generation. For the three selected floats, we computed the average $O_2$ accuracy for each float lifetime (Supplementary Table 1). The accuracies of these four Argo parameters (P, T, S, $O_2$) were taken as uncertainties to estimate the overall $C_{ant}$ uncertainty. The uncertainties of CONTENT are calculated from the standard deviation of the input variables to their respective weighted mean and the mismatch of the four carbonate system variables with respect to their weighted mean (Bittig, Steinhoff, et al., 2018). The uncertainties of CONTENT provide uncertainties that are adapted to the local conditions, and additionally includes the carbonate system description's consistency. The uncertainties of ESPER_NN are based on the root mean squared errors of all predicted variables and these uncertainties depend on depth and salinity (Carter et al., 2021).

To estimate the overall $C_{ant}$ uncertainty, we generated 100 input variables fields following the Monte Carlo method (Metropolis & Ulam, 1949). The generated fields are randomly computed via the formula (Eq. 3):

$$X - \sigma_X \leq X_{MC} \leq X + \sigma_X \qquad (3)$$

where $X_{MC}$ is the new input variable generated via the Monte Carlo method, X is the original input variable and $\sigma_X$ is the error associated with the input variable (Table 1). From these 100 new input variable fields, we calculate 100 $C_{ant}$ fields and compute the standard deviation between these fields. The standard deviation fluctuates between ±5.4 µmol $kg^{-1}$ and ±10.2 µmol $kg^{-1}$ with an overall average of ±5.9 µmol $kg^{-1}$. It can be surprising that the mean $C_{ant}$ uncertainty is lower than the mean uncertainty of DIC (> 10 µmol $kg^{-1}$). However, $C_{ant}$ concentrations are driven by DIC and $O_2$. These two variables are anti-correlated and when $O_2$ increases, usually, DIC decreases due to several biogeophysical mechanisms (Louanchi et al., 2001). Consequently, there is a negative correlation between DIC and $O_2$ uncertainties, meaning that DIC uncertainties are partially compensated by $O_2$ uncertainties in the computation of $C_{ant}$ estimates. This compensation explains the lower $C_{ant}$ uncertainties compared to DIC uncertainties.

**Supplementary Table 1: Uncertainties on the input variables to calculate $C_{ant}$ for the 3 Argo floats.** The uncertainties of pressure, potential temperature, salinity and oxygen after delayed-mode correction are provided by the Argo program. The uncertainties of silicate, nitrate and phosphate correspond to the mean ESPER_NN uncertainties (Carter et al., 2021). The uncertainties of alkalinity and dissolved inorganic carbon represent the mean CONTENT uncertainties (Bittig, Steinhoff, et al., 2018). See text above for more explanations.

| | 5904988 | 6901023 | 6901026 |
|---|---|---|---|
| Pressure (dbar) | ±2.40 | ±2.40 | ±2.40 |
| Pot. temp. (°C) | ±0.002 | ±0.002 | ±0.002 |
| Salinity (psu) | ±0.01 | ±0.01 | ±0.01 |
| Oxygen (μmol $kg^{-1}$) | ±3.50 | ±3.10 | ±3.10 |
| Silicate (μmol $kg^{-1}$) | ±1.72 | ±1.48 | ±1.46 |
| Nitrate (μmol $kg^{-1}$) | ±0.65 | ±0.57 | ±0.56 |
| Phosphate (μmol $kg^{-1}$) | ±0.057 | ±0.053 | ±0.051 |
| Alkalinity (μmol $kg^{-1}$) | ±11.03 | ±10.72 | ±10.80 |
| Dissolved inorganic carbon (μmol $kg^{-1}$) | ±10.66 | ±10.33 | ±10.44 |

# Supplementary References


Bittig, H. C., Fiedler, B., Fietzek, P., & Körtzinger, A. (2015). Pressure Response of Aanderaa and Sea-Bird Oxygen Optodes. *Journal of Atmospheric and Oceanic Technology*, *32*(12), 2305–2317. https://doi.org/10.1175/JTECH-D-15-0108.1

Bittig, H. C., Körtzinger, A., Neill, C., Van Ooijen, E., Plant, J. N., Hahn, J., Johnson, K. S., Yang, B., & Emerson, S. R. (2018). Oxygen optode sensors: Principle, characterization, calibration, and application in the ocean. *Frontiers in Marine Science*, *4*, 429.

Bittig, H. C., Steinhoff, T., Claustre, H., Fiedler, B., Williams, N. L., Sauzède, R., Körtzinger, A., & Gattuso, J.-P. (2018). An alternative to static climatologies: Robust estimation of open ocean $CO_2$ variables and nutrient concentrations from T, S, and $O_2$ data using Bayesian neural networks. *Frontiers in Marine Science*, *5*, 328.

Carter, B. R., Bittig, H. C., Fassbender, A. J., Sharp, J. D., Takeshita, Y., Xu, Y.-Y., Álvarez, M., Wanninkhof, R., Feely, R. A., & Barbero, L. (2021). New and updated global empirical seawater property estimation routines. *Limnology and Oceanography: Methods*, *19*(12), 785–809.

Faghmous, J. H., Frenger, I., Yao, Y., Warmka, R., Lindell, A., & Kumar, V. (2015). A daily global mesoscale ocean eddy dataset from satellite altimetry. *Scientific Data*, *2*(1), 1–16.

García-Ibáñez, M. I., Zunino, P., Fröb, F., Carracedo, L. I., Ríos, A. F., Mercier, H., Olsen, A., & Pérez, F. F. (2016). Ocean acidification in the subpolar North Atlantic: Rates and mechanisms controlling pH changes. *Biogeosciences*, *13*(12), 3701–3715.

Gordon, C., Fennel, K., Richards, C., Shay, L. K., & Brewster, J. K. (2020). Can ocean community production and respiration be determined by measuring high-frequency oxygen profiles from autonomous floats? *Biogeosciences*, *17*(15), 4119–4134. https://doi.org/10.5194/bg-17-4119-2020

Grégoire, M., Garçon, V., Garcia, H., Breitburg, D., Isensee, K., Oschlies, A., Telszewski, M., Barth, A., Bittig, H. C., Carstensen, J., Carval, T., Chai, F., Chavez, F., Conley, D., Coppola, L., Crowe, S., Currie, K., Dai, M., Deflandre, B., … Yasuhara, M. (2021). A Global Ocean Oxygen Database and Atlas for Assessing and Predicting Deoxygenation and Ocean Health in the Open and Coastal Ocean. *Frontiers in Marine Science*, *8*, 724913. https://doi.org/10.3389/fmars.2021.724913

Gruber, N., Clement, D., Carter, B. R., Feely, R. A., Van Heuven, S., Hoppema, M., Ishii, M., Key, R. M., Kozyr, A., Lauvset, S. K., & others. (2019). The oceanic sink for anthropogenic $CO_2$ from 1994 to 2007. *Science*, *363*(6432), 1193–1199.

Gruber, N., Sarmiento, J. L., & Stocker, T. F. (1996). An improved method for detecting anthropogenic $CO_2$ in the oceans. *Global Biogeochemical Cycles*, *10*(4), 809–837. https://doi.org/10.1029/96GB01608

Louanchi, F., Ruiz-Pino, D. P., Jeandel, C., Brunet, C., Schauer, B., Masson, A., Fiala, M., & Poisson, A. (2001). Dissolved inorganic carbon, alkalinity, nutrient and oxygen seasonal and interannual variations at the Antarctic Ocean JGOFS-KERFIX site. *Deep Sea Research Part I: Oceanographic Research Papers*, *48*(7), 1581–1603. https://doi.org/10.1016/S0967-0637(00)00086-8

Metropolis, N., & Ulam, S. (1949). The Monte Carlo Method. *Journal of the American Statistical Association*, *44*(247), 335–341.

Racapé, V., Thierry, V., Mercier, H., & Cabanes, C. (2019). ISOW spreading and mixing as revealed by Deep-Argo floats launched in the Charlie-Gibbs fracture zone. *Journal of Geophysical Research: Oceans*, *124*(10), 6787–6808.

Talley, L. D., Pickard, G. L., & Emery, W. J. (Eds.). (2011). *Descriptive physical oceanography: An introduction* (6th ed). Academic Press.

Tanhua, T., Körtzinger, A., Friis, K., Waugh, D. W., & Wallace, D. W. R. (2007). An estimate of anthropogenic $CO_2$ inventory from decadal changes in oceanic carbon content. *Proceedings of the National Academy of Sciences*, *104*(9), 3037–3042. https://doi.org/10.1073/pnas.0606574104

Thierry, V., Bittig, H., & The Argo-BGC team. (2021). *Argo quality control manual for dissolved oxygen concentration*.

Vazquez-Rodriguez, M., Touratier, F., Lo Monaco, C., Waugh, D., Padin, X. A., Bellerby, R. G., Goyet, C., Metzl, N., Ríos, A. F., & Pérez, F. F. (2009). Anthropogenic carbon distributions in the Atlantic Ocean: Data-based estimates from the Arctic to the Antarctic. *Biogeosciences*, *6*(3), 439–451.

Wong, A. P. S., Wijffels, S. E., Riser, S. C., Pouliquen, S., Hosoda, S., Roemmich, D., Gilson, J., Johnson, G. C., Martini, K., Murphy, D. J., Scanderbeg, M., Bhaskar, T. V. S. U., Buck, J. J. H., Merceur, F., Carval, T., Maze, G., Cabanes, C., André, X., Poffa, N., … Park, H.-M. (2020). Argo Data 1999–2019: Two Million Temperature-Salinity Profiles and Subsurface Velocity Observations From a Global Array of Profiling Floats. *Frontiers in Marine Science*, *7*, 700. https://doi.org/10.3389/fmars.2020.00700
