# Supplementary material for: Anthropogenic carbon pathways towards the North Atlantic interior revealed by Argo-O2, neural networks and back-calculations
Source: Nat Commun. 2024 Feb 22;15:1630. doi: 10.1038/s41467-024-46074-5 (PMC10884407; doi:10.1038/s41467-024-46074-5)
Supplement: Supplementary file 3 — Reporting Summary [file 41467_2024_46074_MOESM3_ESM.pdf]

nature portfolio

Corresponding author(s): Rémy Asselot (remy.asselot@ifremer.fr)

Last updated by author(s): Jan 5, 2024

Reporting Summary

Nature Portfolio wishes to improve the reproducibility of the work that we publish. This form provides structure for consistency and transparency in reporting. For further information on Nature Portfolio policies, see our Editorial Policies and the Editorial Policy Checklist.

Statistics

For all statistical analyses, confirm that the following items are present in the figure legend, table legend, main text, or Methods section.

n/a	Confirmed	
☐	☒	The exact sample size (*n*) for each experimental group/condition, given as a discrete number and unit of measurement
☐	☒	A statement on whether measurements were taken from distinct samples or whether the same sample was measured repeatedly
☒	☐	The statistical test(s) used AND whether they are one- or two-sided<br>*Only common tests should be described solely by name; describe more complex techniques in the Methods section.*
☐	☒	A description of all covariates tested
☒	☐	A description of any assumptions or corrections, such as tests of normality and adjustment for multiple comparisons
☐	☒	A full description of the statistical parameters including central tendency (e.g. means) or other basic estimates (e.g. regression coefficient) AND variation (e.g. standard deviation) or associated estimates of uncertainty (e.g. confidence intervals)
☒	☐	For null hypothesis testing, the test statistic (e.g. *F*, *t*, *r*) with confidence intervals, effect sizes, degrees of freedom and *P* value noted<br>*Give P values as exact values whenever suitable.*
☒	☐	For Bayesian analysis, information on the choice of priors and Markov chain Monte Carlo settings
☒	☐	For hierarchical and complex designs, identification of the appropriate level for tests and full reporting of outcomes
☒	☐	Estimates of effect sizes (e.g. Cohen's *d*, Pearson's *r*), indicating how they were calculated

Our web collection on statistics for biologists contains articles on many of the points above.

Software and code

Policy information about availability of computer code

Data collection	The data have been collected with Argo floats, available on the Euro Argo Data Selection platform (https://fleetmonitoring.euro-argo.eu/)
Data analysis	The data analysis has been performed with Matlab and the code is available on demand.

For manuscripts utilizing custom algorithms or software that are central to the research but not yet described in published literature, software must be made available to editors and reviewers. We strongly encourage code deposition in a community repository (e.g. GitHub). See the Nature Portfolio guidelines for submitting code & software for further information.

Data

Policy information about availability of data

All manuscripts must include a data availability statement. This statement should provide the following information, where applicable:

- Accession codes, unique identifiers, or web links for publicly available datasets
- A description of any restrictions on data availability
- For clinical datasets or third party data, please ensure that the statement adheres to our policy

The original Argo data can be downloaded on the Euro Argo Data Selection platform. Our Cant estimates, needed to evaluate the conclusion of the paper, can be downloaded on Zenodo (https://doi.org/10.5281/zenodo.7071614).

Research involving human participants, their data, or biological material

Policy information about studies with human participants or human data. See also policy information about sex, gender (identity/presentation), and sexual orientation and race, ethnicity and racism.

Reporting on sex and gender	Our research didn't involve humans or biological material
Reporting on race, ethnicity, or other socially relevant groupings	Our research didn't involve humans or biological material
Population characteristics	Our research didn't involve humans or biological material
Recruitment	Our research didn't involve humans or biological material
Ethics oversight	Our research didn't involve humans or biological material

Note that full information on the approval of the study protocol must also be provided in the manuscript.

Field-specific reporting

Please select the one below that is the best fit for your research. If you are not sure, read the appropriate sections before making your selection.

☐ Life sciences ☐ Behavioural & social sciences ☒ Ecological, evolutionary & environmental sciences

For a reference copy of the document with all sections, see nature.com/documents/nr-reporting-summary-flat.pdf

Ecological, evolutionary & environmental sciences study design

All studies must disclose on these points even when the disclosure is negative.

Study description	Our study describes the deepening of anthropogenic carbon (Cant) in the subpolar North Atlantic gyre. To do so, we combine Argo data, neural networks and a back-calculation method. Our results demonstrate that Cant follows a stepwise deepening along its way through the North Atlantic subpolar gyre.
Research sample	Our data come from Argo floats that measure temperature, salinity, oxygen every 10 days over the top 2000 dbar of the water column. These Argo data are used as inputs for neural networks. The outputs of the neural networks plus the original Argo data are used to estimate Cant via a back claculation method.
Sampling strategy	We selected the Argo-O2 floats that (1) followed the cyclonic pathway of the subpolar North Atlantic gyre, (2) had a lifetime longer than 3 years, and (3) crossed the A25 OVIDE hydrographic section. Three floats matched these criteria.
Data collection	The Argo program is a public program and the data are freely available on https://fleetmonitoring.euro-argo.eu/
Timing and spatial scale	Our data span from July 2012 to April 2018 and are located in the subpolar North Atlantic gyre (47-65°N; 15-65°W).
Data exclusions	None of the data were excluded
Reproducibility	The original Argo data are freely available at https://fleetmonitoring.euro-argo.eu/<br>The Matlab code to reproduce our results are available on demand.
Randomization	Not applicable
Blinding	Not applicable

Did the study involve field work? ☐ Yes ☒ No

Reporting for specific materials, systems and methods

We require information from authors about some types of materials, experimental systems and methods used in many studies. Here, indicate whether each material, system or method listed is relevant to your study. If you are not sure if a list item applies to your research, read the appropriate section before selecting a response.

Materials & experimental systems

n/a	Involved in the study
☒	☐ Antibodies
☒	☐ Eukaryotic cell lines
☒	☐ Palaeontology and archaeology
☒	☐ Animals and other organisms
☒	☐ Clinical data
☒	☐ Dual use research of concern
☒	☐ Plants

Methods

n/a	Involved in the study
☒	☐ ChIP-seq
☒	☐ Flow cytometry
☒	☐ MRI-based neuroimaging
